# Suzuki–Miyaura cross coupling is not an informative reaction to demonstrate the performance of new solvents

**DOI:** 10.3762/bjoc.16.89

**Published:** 2020-05-13

**Authors:** James Sherwood

**Affiliations:** 1 Green Chemistry Centre of Excellence, Department of Chemistry, University of York, Heslington, YO10 5DD, UK

**Keywords:** cross coupling, green solvents, palladium, solvent selection, Suzuki reaction

## Abstract

The development and study of new solvents has become important due to a proliferation of regulations preventing or limiting the use of many conventional solvents. In this work, the suitability of the Suzuki–Miyaura reaction to demonstrate the usefulness of new solvents was evaluated, including Cyrene™, dimethyl isosorbide, ethyl lactate, 2-methyltetrahydrofuran (2-MeTHF), propylene carbonate, and γ-valerolactone (GVL). It was found that the cross coupling is often unaffected by the choice of solvent, and therefore the Suzuki–Miyaura reaction provides limited information regarding the usefulness of any particular solvent for organic synthesis.

## Findings

The objective of this work was to reveal if there is a relationship between the productivity of Suzuki–Miyaura cross couplings and the properties of the solvent, and whether this could be used to justify solvent selection. The choice of solvent is one variable that dictates reaction rate, selectivity, equilibria, solubility, and ultimately product yield. If there is an observable change in reaction performance correlating to one or more solvent properties (often polarity), then it is possible to identify and implement an optimum solvent. Suzuki–Miyaura cross coupling is the premier method of palladium catalysed carbon–carbon bond formation, making it an obvious case study to validate the performance of novel solvents [1–7]. The polarity of the solvent is known to determine the structure and activity of catalytic intermediates, the rate determining step, and stereochemistry (where applicable) of Suzuki–Miyaura cross couplings [8]. Despite this, the reaction is generally tolerant of a wide range of solvents (often an ether or amide solvent is used, and water is a common co-solvent). This calls into question the benefits of using Suzuki–Miyaura cross coupling as a test of new solvents, regardless of how vital the reaction is.

**Scheme 1:**
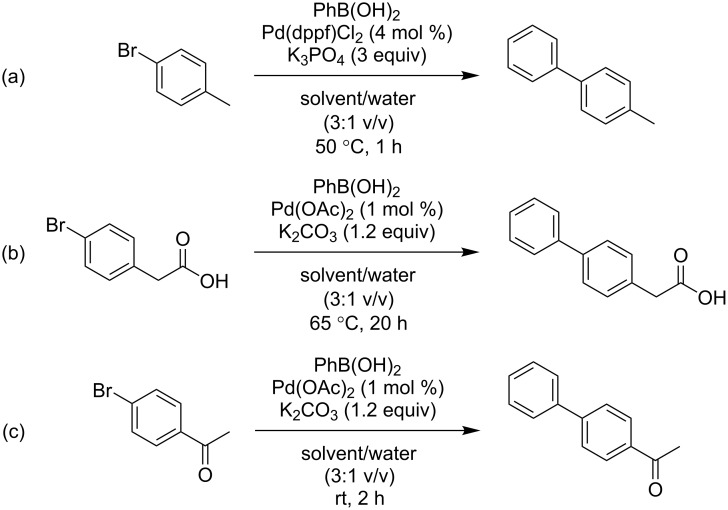
(a) Case study 1, reaction of 4-bromotoluene; (b) case study 2, reaction of 4-bromophenylacetic acid; (c) case study 3, reaction of 4’-bromoacetophenone.

Three variations of the Suzuki–Miyaura cross-coupling protocol were performed. Each case study is a transformation of phenylboronic acid (1.2 molar equivalents) under different conditions (see Scheme 1), but all using 1 part water to 3 parts organic solvent (by volume) and 0.6 mmol (1 equivalent) of an aryl bromide. The solvent screening included twelve solvents. The following eminent green and bio-based solvents were included in the study to assess their ability to substitute conventional solvents: Cyrene™ [3], and its alcohol equivalent levoglucosanol [9], ethyl lactate [10], 2-methyltetrahydrofuran (2-MeTHF) [11], γ-valerolactone (GVL) [12], dimethyl isosorbide (DMI) [6], and propylene carbonate [7]. This study compares solvents under the same conditions to offer a fair comparison. Additional solvents were included to ensure a range of polarities were investigated (see Supporting Information File 1). In each case study, conversion to the desired product was measured by ^1^H NMR spectroscopy (see Supporting Information File 1, Figure S4). The results are summarised in Table 1. No evidence of significant hydrodehalogenation or other unintended reactions was observed throughout unless noted subsequently.

**Table 1:** Conversions in three Suzuki–Miyaura reactions.^a^

solvent	greenness ranking	case study 1	case study 2	case study 3

NMP	hazardous (reprotoxicity)	85%	98%	100%
toluene	problematic (health and safety issues)	94%	100%	42%
butanone	recommended	92%	92%	30%
2-propanol	recommended	81%	100%	100%
ethyl acetate	recommended	76%	100%	28%
ethyl lactate	problematic (causes serious eye damage)	75%	81%	73%
Cyrene™	problematic (low volatility)	83%	2%	5%
levoglucosanol	lacking data (low volatility)	77%	64%	82%
DMI	problematic (low volatility)	96%	63%	74%
propylene carbonate	problematic (low volatility)	81%	100%	98%
GVL	problematic (low volatility)	87%	98%	36%
2-MeTHF	problematic (health and safety issues)	79%	100%	16%

^a^Greenness ranking is taken from the CHEM21 solvent selection guide.

The first case study was adapted from that developed by Watson and co-workers [3,6]. The desired coupling is of 4-bromotoluene to produce 4-phenyltoluene, assisted by the inclusion of the bis(diphenylphosphino)ferrocene ligand and 3 equivalents of base (Scheme 1a). In this work the proportion of water added is less than that previously optimised for reactions in Cyrene™ [3], and more than that previously optimised for reactions in DMI [6]. In case study 1, the majority of solvents resulted in conversion to the product in the range of 75–85% after 1 hour. The highest conversion of 96% was obtained in DMI, but overall it is fair to conclude the reaction quickly reaches good conversions with little apparent influence from the solvent.

The second case study transformed 4-bromophenylacetic acid into felbinac, a nonsteroidal anti-inflammatory drug (Scheme 1b) [13]. Here, palladium acetate without an auxiliary ligand was used for a pre-catalyst and the base changed to potassium carbonate. Reaction conditions of 20 hours at 65 °C were decided after observing 11% conversion after 2 hours and 33% after 20 hours in NMP at room temperature. The majority of solvents provided conversions in excess of 90%. In the case of Cyrene™, its instability towards inorganic bases is presumably the reason for the very low conversion (2%). During one run the reaction mixture did solidify, as has been reported previously for various chemistries in Cyrene™ under basic conditions [14].

The third case study was a coupling of 4-bromoacetophenone (Scheme 1c) using the same pre-catalyst and base as in case study 2. In this example the reaction proceeds at room temperature, but now the conversion to the product varies considerably between solvents. Reactions in *N*-methyl-2-pyrrolidone (NMP) and 2-propanol (IPA) resulted in complete conversion, and the product could be isolated by crystallisation from diethyl ether. Propylene carbonate also provided excellent conversion to the product (98%). The alcohol functionalised solvents outperformed their aprotic analogues, while Cyrene™, GVL, and 2-MeTHF were poor solvents. Replacing potassium carbonate with triethylamine, the conversion in Cyrene™ rose slightly to 10%. Despite the variation between experiments no discernible correlation between any solvent properties and the observed conversions was found.

The results achieved in DMI across the three case studies typify the lack of an obvious solvent effect. In one instance DMI is the best performing solvent (case study 1), then the worst aside from the reactive Cyrene™ (case study 2), and then somewhere in between (case study 3). To demonstrate that not even case study 3 is robust enough to definitively establish a measurement of solvent performance in Suzuki–Miyaura cross couplings, a short optimisation study was conducted to improve the conversion to 4-phenylacetophenone in 2-MeTHF (originally 16%). Reducing the water content to an 18:1 v/v ratio and increasing the excess of base to 3 equivalents and catalyst to 5 mol % was found to be beneficial, as was a higher reaction temperature of 65 °C. These conditions produced a conversion of 79% after 4 hours in 2-MeTHF. This is an indication of the weak influence of the solvent compared to the impact of the reaction temperature, and the choice and quantity of catalyst and base.

Given the broad choice of solvents available, what is left to decide is the most benign solvent that should be preferred for conducting Suzuki–Miyaura reactions. Table 1 lists the greenness rating from the CHEM21 solvent selection guide (except for levoglucosanol which lacks the necessary data) [15]. The ‘recommended’ solvent with a high performance across the three case studies is IPA, known as a robust solvent for Suzuki–Miyaura type cross couplings [16–18]. However, it is also worth noting that the ‘problematic’ designation of Cyrene™, DMI, propylene carbonate, and GVL is due to their high boiling points placing a high energy demand on recovery by distillation. If recovery has been considered and deemed infeasible, then propylene carbonate in particular should also be considered given its superior hazard profile compared to IPA. However, caution is advised in the presence of nucleophilic reagents, as this has previously been reported to cause ring opening of propylene carbonate during Suzuki–Miyaura cross couplings [7]. In this work no decomposition of propylene carbonate was identified. Using only water as a solvent is also appealing from a green chemistry perspective if the water can be reused. To this end, micellar chemistry is appropriate for cross couplings [19]. Residual water also assists ‘solvent-free’ methods [20].

In summary, the Suzuki–Miyaura reaction is a fantastically versatile and industrially important reaction [21–22], and excels in a variety of reaction media. On the evidence of this study, it can be concluded that the Suzuki–Miyaura reaction is not an informative case study for solvent effects and cannot reliably validate the benefits of one particular solvent. This is because catalysts and conditions can be chosen to promote high conversions regardless of the properties of the solvent. Additionally, the diverse properties of high performance solvents across Suzuki–Miyaura reactions means it is hard to discern what are the requisite qualities of the reaction medium (if any) that encourage the desired cross coupling. Specific mechanistic studies whereby the rate limiting step or mechanism changes according to the solvent remain a valid pursuit, as does measuring the palladium contamination in products [23]. However, the works of Watson [24], Denmark [25], and others [26–27], have already elucidated many of the key fundamental principles of boron and palladium speciation and the role of the base in the Suzuki–Miyaura reaction.

For researchers developing safer solvents, the Mizoroki–Heck reaction is a more suitable cross-coupling methodology to demonstrate solvent performance [28]. The reaction kinetics of Mizoroki–Heck reactions have a strong dependence on the dipolarity of the reaction medium, and the rate determining step can be controlled by the equivalents of ligand added, thereby eliminating one variable [8]. Reprotoxic solvents such as *N*,*N*-dimethylformamide (DMF) are routinely used in the Mizoroki–Heck reaction and hence there is also a motivation to investigate safer alternative solvents that the Suzuki–Miyaura reaction lacks.

If researchers are still compelled to study the utility of solvents in the Suzuki–Miyaura reaction, I encourage future studies to be directed at challenging substrates that correspond to commercially important products (e.g., enantiopure pharmaceuticals, polymeric materials) and if a substrate screening should follow, the protocol established by Collins and Glorius is effective [29]. For the development of new catalysts, it is preferable to work with a benign solvent such as aqueous IPA from the outset [30]. This is because late-stage solvent screens rarely reveal a superior solvent due to the catalyst having already been optimised to work in the original solvent, which may have been chosen only for ease of removal (e.g., the volatile but suspected carcinogen dichloromethane) or the high solubility of organic and inorganic reagents (e.g., the reprotoxic DMF).

## Supporting Information

File 1 Synthetic procedures and calculation of reaction conversions and solvent polarity data.
