# Supplementary material for: Suzuki–Miyaura cross coupling is not an informative reaction to demonstrate the performance of new solvents
Source: Beilstein J Org Chem. 2020 May 13;16:1001–5. doi: 10.3762/bjoc.16.89 (PMC7237806; doi:10.3762/bjoc.16.89)
Supplement: File 1 — Synthetic procedures and calculation of reaction conversions and solvent polarity data. [file Beilstein_J_Org_Chem-16-1001-s001.pdf]

BEILSTEIN JOURNAL OF ORGANIC CHEMISTRY

Supporting Information

for

Suzuki–Miyaura cross coupling is not an informative reaction to demonstrate the performance of new solvents

James Sherwood

Beilstein J. Org. Chem. **2020,** *16,* 1001–1005. doi:10.3762/bjoc.16.89

Synthetic procedures and calculation of reaction conversions and solvent polarity data

License and Terms: This is a supporting information file under the terms of the Creative Commons Attribution License (http://creativecommons.org/licenses/by/4.0). Please note that the reuse, redistribution and reproduction in particular requires that the authors and source are credited.
The license is subject to the *Beilstein Journal of Organic Chemistry* terms and conditions: (https://www.beilstein-journals.org/bjoc)

Synthetic procedures

Conditions and quantities summarised in Table S1.

Table S1: Summary of the variable conditions across the case studies.

variable	case study 1	case study 2	case study 3
aryl bromide	4-bromotoluene	4-bromophenyl acetic acid	4'-bromo-acetophenone
pre-catalyst	$Pd(dppf)Cl_2$ (4 mol %)	$Pd(OAc)_2$ (1 mol %)	$Pd(OAc)_2$ (1 mol %)
base	K_3PO_4 (3 equiv)	K_2CO_3 (1.2 equiv)	K_2CO_3 (1.2 equiv)
temperature	50 °C	65 °C	ambient
duration	1 hour	20 hours	2 hours

4-Phenyltoluene. To 4-bromotoluene (0.103 g, 0.6 mmol) was added phenylboronic acid (0.0878 g, 1.2 equiv.), [1,1′-bis(diphenylphosphino)ferrocene]dichloro-palladium(II)-DCM complex (0.0196 g, 4 mol %), potassium phosphate (0.382 g, 3 equiv), water (0.6 mL) and the chosen solvent (1.8 mL). The reaction mixture was stirred for 1 hours at 50 °C, and then diluted with diethyl ether (20 mL) and washed with water (3 × 20 mL). The organic phase was concentrated and the resulting crude solid analysed by ^{1}H NMR spectroscopy (Figure S1).

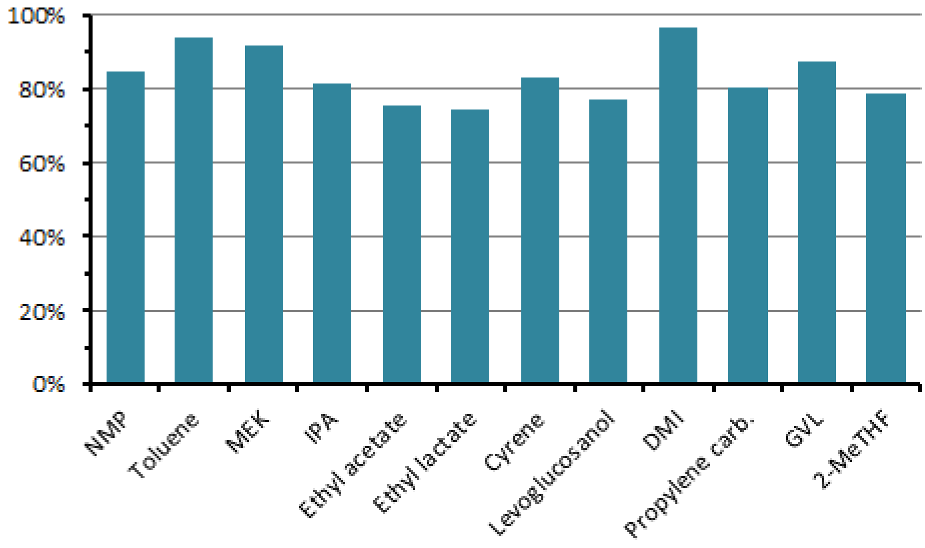

Figure S1: 4-Bromotoluene conversion and isolated mass.

Felbinac. To 4-bromophenyl acetic acid (0.129 g, 0.6 mmol) was added phenylboronic acid (0.0878 g, 1.2 equiv), palladium acetate (0.0013 g, 1 mol %), potassium carbonate (0.0995 g, 1.2 equiv), water (0.6 mL) and the chosen solvent (1.8 mL). The reaction mixture was stirred for 20 hours at 65 °C, and then allowed to cool to the ambient temperature. The reaction mixture was diluted with water (10 mL), acidified with hydrochloric acid to produce a precipitate, and extracted with dichloromethane (2 × 15 mL). The organic phase was concentrated and the resulting crude solid analysed by ^{1}H NMR spectroscopy (Figure S2).

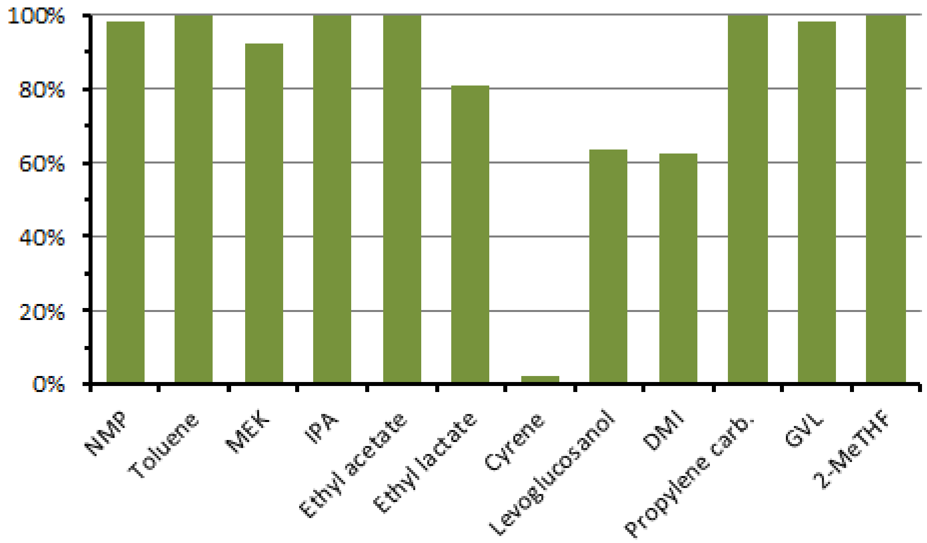

Figure S2: 4-Bromophenylacetic acid conversion and isolated mass.

Biphenyl methyl ketone. To 4-bromoacetophenone (0.119 g, 0.6 mmol) was added phenylboronic acid (0.0878 g, 1.2 equiv), palladium acetate (0.0013 g, 1 mol %), potassium carbonate (0.0995 g, 1.2 equiv), water (0.6 mL) and the chosen solvent (1.8 mL). The reaction mixture was stirred for 2 hours at the ambient temperature, and then diluted with diethyl ether (20 mL) and washed with water (2 × 20 mL). The organic phase was concentrated and the resulting crude solid analysed by ^{1}H NMR spectroscopy (Figure S3).

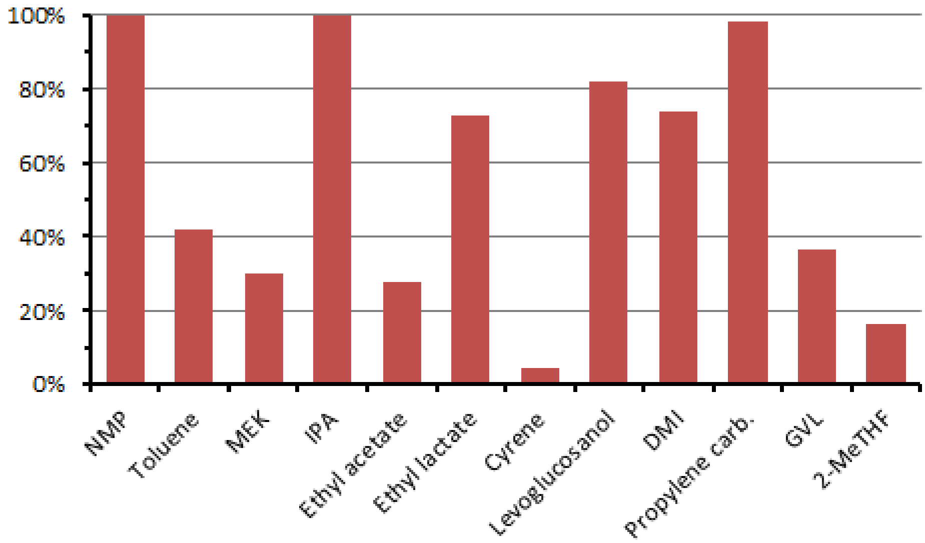

Figure S3: 4-Bromoacetophenone conversions.

Calculation of reaction conversions

The conversion from bromoarene to biphenyl product was calculated by identifying peaks in ^{1}H NMR spectra that correspond to the same functionality before and after coupling (i.e. a methyl or methylene group). The signal moves downfield upon replacing a bromide with a phenyl group for all three case studies (Figure S4). The integral of the product signal was divided by the sum of the reactant and product signals to produce an estimate of conversion.

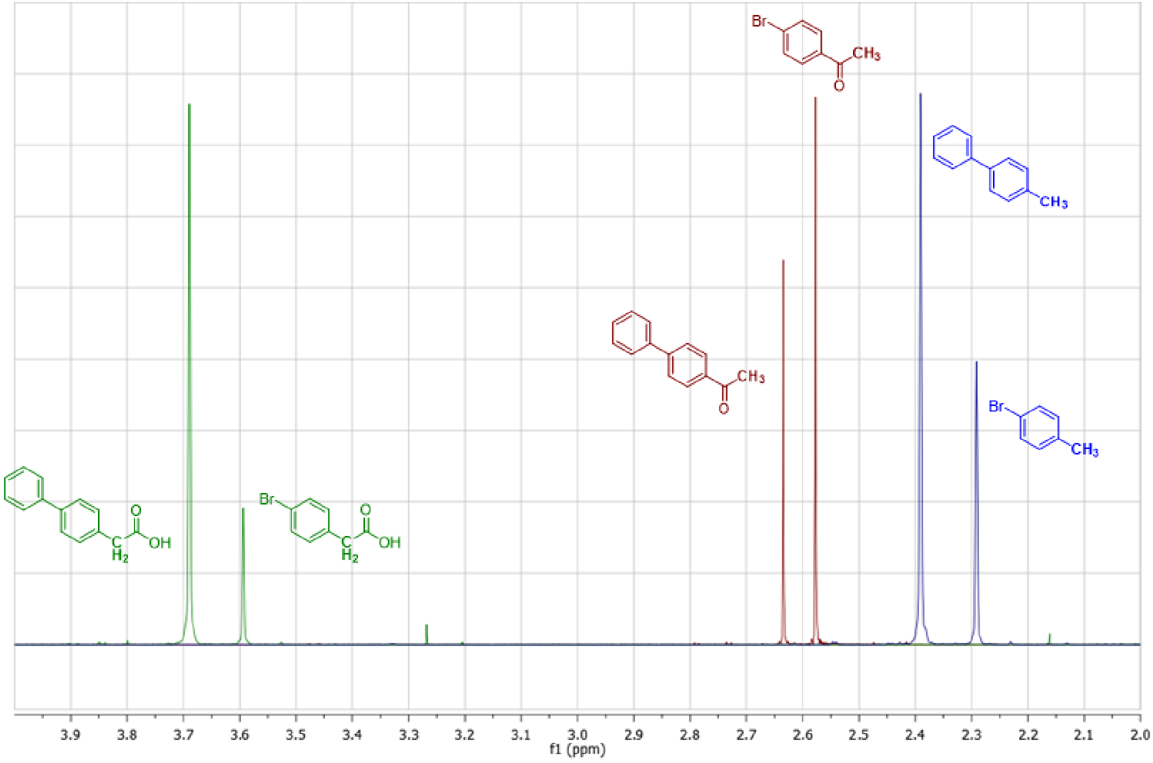

Figure S4: Example spectra for calculating conversions (overlaid, 4–2 ppm range). Key (left to right): Green, Felbinac synthesis in ethyl lactate; red, synthesis of *p*-phenylacetophenone in toluene; blue, *p*-phenyltoluene in butanone.

Solvent polarity data

The solvents chosen for this study have a range of polarities, as is necessary to discern solvent effects. Hansen solubility parameters describe solubility, and Kamlet–Abboud–Taft solvatochromic parameters correlate to reaction rates and equilibria (Figure S5). The majority of solvents were selected because they are of interest as greener or bio-based solvents. In three instances an alcohol analogue of an aprotic solvent was included to investigate any influence by hydrogen bond donation: 2-propanol (IPA) and butanone (MEK, chosen instead of acetone because of its higher boiling point); ethyl lactate and ethyl acetate; Cyrene™ and levoglucosanol. Toluene and *N*-methyl pyrrolidone (NMP) were also included as known solvents for this category of reaction but with dissimilar physical properties, extending the range of polarities covered. Overall the solvent set includes solvents miscible and non-miscible with water, and a range of functional groups (amide, ether, ketone, ester, alcohol, carbonate, and arene). Low polarity alkanes (with small π^* values) are not included to avoid solubility becoming a factor in determining conversions, and they are uncommon as solvents in Suzuki–Miyaura cross-couplings. Low polarity, strong hydrogen bond accepting (high β) amine solvents were excluded to avoid formation of ammonium hydroxide with water and creating non-comparable results due to a change in base concentration.

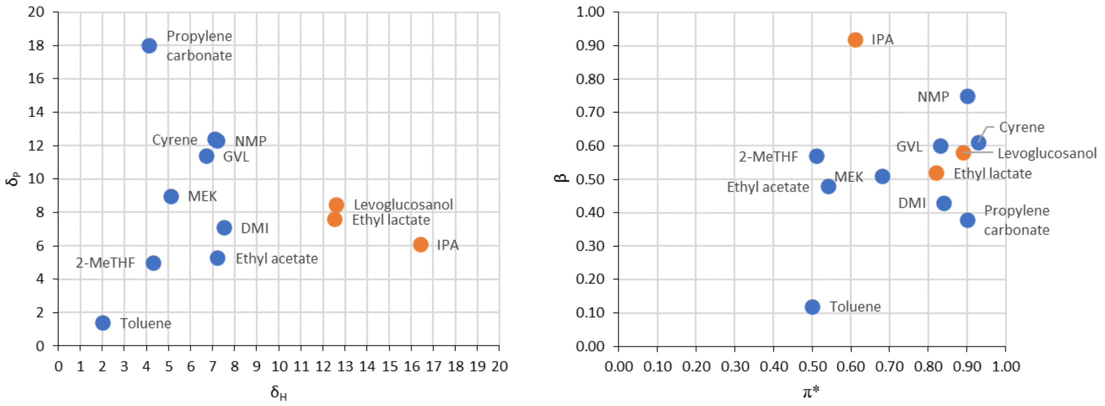

Figure S5: Hansen solubility parameters of dipolarity (δ_P /MPa½) and hydrogen bonding (δ_H /MPa½) (left) [1], and Kamlet–Abboud–Taft solvatochromic parameters of hydrogen bond accepting ability (β) and dipolarity (π*) (right) [2-10], of protic solvents (orange) and aprotic solvents (blue).

References

1. *HSPiP*, version 5.2.06; Abbott, S; Yamamoto, H.: 2015.
2. Clark, J. H.; Macquarrie, D. J.; Sherwood, J. *Green Chem.* **2012,** *14,* 90-93.
3. Jessop, P. G.; Jessop, D. A.; Fu, D.; Phan, L. *Green Chem.* **2012,** *14,* 1245.
4. Kamlet, M. J.; Taft, R. W. *J. Am. Chem. Soc.* **1976,** *98,* 377.
5. Mouret, A.; Leclercq, L.; Mühlbauer, A.; Nardello-Rataj, V. *Green Chem.* **2014,** *16,* 269.
6. Parker, H. L.; Sherwood, J.; Hunt, A. J.; Clark, J. H. *ACS Sustainable Chem. Eng.* **2014,** *2,* 1739.
7. Sherwood, J. Bio-Based Solvents for Organic Synthesis. Ph.D. Thesis, University of York, U.K., 2013.
8. Sherwood, J.; De bruyn, M.; Constantinou, A.; Moity, L.; McElroy, C. R.; Farmer, T. J.; Duncan, T.; Raverty, W.; Hunt, A. J.; Clark, J. H. *Chem. Commun.* **2014,** *50,* 9650.
9. Sherwood, J.; Granelli, J.; McElroy, C. R.; Clark, J. H. *Molecules* **2019,** *24,* 2209.
10. Taft R. W.; Kamlet, M. J. *J. Am. Chem. Soc.* **1976,** *98,* 2886.
